# Antioxidant Capacity and Antiplatelet Activity of Aqueous Extracts of Common Bean (*Phaseolus vulgaris* L.) Obtained with Microwave and Ultrasound Assisted Extraction

**DOI:** 10.3390/plants11091179

**Published:** 2022-04-27

**Authors:** Lyanne Rodríguez, Andrea Plaza, Diego Méndez, Basilio Carrasco, Francisca Tellería, Iván Palomo, Eduardo Fuentes

**Affiliations:** 1 Centro de Estudios en Alimentos Procesados (CEAP), CONICYT Programa Regional, Gore Maule R0912001, Casilla 1007, Talca 3480094, Chile; lyannerodriguez89@gmail.com (L.R.); aplaza@ceap.cl (A.P.); diego.mendez@utalca.cl (D.M.); bcarrasco@ceap.cl (B.C.); 2 Thrombosis Research Center, Medical Technology School, Department of Clinical Biochemistry and Immunohaematology, Faculty of Health Sciences, Universidad de Talca, Talca 3480094, Chile; f.tellerialopez@gmail.com

**Keywords:** antiplatelet, antioxidant, common bean, ultrasonic assisted extraction, microwave assisted extraction

## Abstract

*Phaseolus vulgaris* L. has beneficial effects on several chronic non-communicable diseases (e.g., cardiovascular diseases) related to oxidative stress. This redox state may influence platelet activation and aggregation; which is crucial in thrombus formation. In this work, the antiplatelet and antioxidant potential of aqueous extracts obtained by green processes, microwave-assisted extraction and ultrasound-assisted extraction, from 25 landraces of common beans were investigated. Phenol content and antioxidant potential were determined using the Folin-Ciocalteu method, total monomeric anthocyanin and ORAC assay, respectively. The antiplatelet potential of the extracts was explored by turbidimetry. Microwave extraction showed higher phenol content and antioxidant activity in most extracts. Soja landrace extract obtained by microwave-assisted extraction showed higher phenol content and antioxidant activity (893.45 ± 87.30 mg GAE/g and 35,642.85 ± 2588.88 ORAC μmolTE/g, respectively). Although most of the extracts obtained by microwave-assisted extraction showed antiplatelet activity, the extract of Hallado Aleman landrace obtained by ultrasound-assisted extraction (IC_50_ = 0.152 ± 0.018 mg/mL) had the highest antiplatelet potential. The extraction method, MAE and UAE, influences the biological potential of the beans, specifically the antiplatelet activity and antioxidant activity. The functional value of this legume for direct consumption by the population was evidenced, as well as its inclusion in food formulations.

## 1. Introduction

Cardiovascular diseases (CVD) account for most chronic non-communicable diseases deaths, (17.9 million people annually), followed by cancers (9.0 million), respiratory diseases (3.9 million), and diabetes (1.6 million) [1]. Oxidative stress is a factor of great importance in the progression of these chronic diseases [2,3]. In the case of CVD, oxidative stress and platelet activation are often closely related. Thus the increase in Reactive oxygen species (ROS) in the circulation exposes the platelets to an activating medium, promoting the change of platelet phenotype to a pro-adhesive one and aggregation, which in turn leads to thromboembolic propensity [4,5].

Given the high prevalence and pathophysiology of CVD; exists an increase in demand for food sources that contribute to its prevention [6,7]. In recent years, the study of bioactive compounds derived from fruits and vegetables capable of generating antiplatelet activity, as well as their mechanism of action, has been increasing. In this way, it has been identified that mango (*Mangifera indica*), maqui (*Aristotelia chilensis*), guava (*Psidium guajava*), turmeric (*Curcuma longa*), and tomato, among others, have antiplatelet potential, mainly associated with a high content of phenolic compounds [8,9,10].

The common bean (*Phaseolus vulgaris* L.) is a major grain legume consumed worldwide and, depending on the type of cultivar, are mainly composed of protein (9 to 50 g/100 g), carbohydrates (20 to 75 g/100 g), lipids (1.5 to 25 g/100 g), dietary fiber (14 to 19 g/100 g) [11] as well as been rich in bioactive compounds [12].

Several studies show the broad chemical profile of common beans, where phenolic acids stand out (p-coumaric acid, synaptic acid, ferulic acid, feruloyl aldaric acid, p- hydroxybenzoic acid, protocatechuic acid, vanillic acid and syringic acid), isoflavonoids (genistein, daidzein, glycitein, formononetin), flavanones (naringenin and hesperetin), anthocyanins (delphinidin, petunidin, malvidin and cyanidin, pelargonidin), sugars, fatty acids, and tocopherols [13,14], among many other compounds that have been reported by our group previously [13]. The content of bioactive compounds varies according to the landrace [14,15,16] and extraction technique [17,18]. Table S1 shows the content of bioactive compounds for different landraces and the extraction technique used.

Some of the problems presented by conventional extraction technologies are long extraction times, the use of expensive and high-purity solvents, evaporation of large amounts of solvent due to the high temperatures used, low extraction selectivity, and thermal decomposition of thermolabile compounds [19,20]. To overcome these problems, unconventional techniques emerged, also called green or clean [18,20,21]. Green extraction methods have been developed using modern technologies, in which less or no organic solvents are used to minimize health and environmental impacts and maximize the yield of desired polyphenols through selective extraction. A definition of green extraction of natural products is “Green extraction is based on the discovery and design of extraction process which will reduce energy consumption, allows use of alternative solvents and renewable natural products, and ensure a safe and high-quality extract/product” [22,23].

The most widely used green processes are ultrasound-assisted extraction (UAE), supercritical fluids (SCFs), microwave-assisted extraction (MAE), and pressurized liquid extraction (PLE) [24,25].

Generally speaking, microwave-assisted extraction (MAE) and ultrasound-assisted extraction (UAE) are promising techniques when compared to the conventional ones, because they are simple and accessible methods that give high yields in a shorter time with minimal solvent and they can be used with different plant materials, including common beans [18].

MAE is carried out using non-ionizing microwave radiation (100–900 W) to the hydrated sample. The sample is subjected to the irradiation of microwave waves causing the movement of the molecules by migration of ions and dipoles that contribute to the increase in temperature, inducing cell wall destruction, and then facilitating the diffusion of compounds from the matrix to the solvent [26]. Sutivisedsak et al. showed that MAE is an effective method for the extraction of phytochemical phenolics in 8 types of North American beans. The content of total phenols using MAE and water as solvent at 100 °C was two to three times higher than the values obtained by conventional extraction [16].

UAE offers substantial advantages over conventional methods compared to traditional extraction methods since the propagation of ultrasound waves (over 20 kHz) creates a breakdown of cellular material by the cavitation phenomenon, which generates a better penetration of solvents in cellular materials and reduces the duration of the process [27,28]. Indeed, ultrasound-assisted extraction technology can potentially improve the extraction of components such as polyphenols, anthocyanins, aromatics, polysaccharides, oils, and functional compounds when is used as a pretreatment in a processing unit [29]. Yang et al., 2019, revealed that UAE exhibits a higher extraction rate of antioxidants from common bean, showing an extraction efficiency seven times higher than conventional solvent extraction. Some of the compounds detected in this study were Protocatechuic acid, catechin, chlorogenic acid, epicatechin, ferulic acid, coumarin, rutin, myricetin, cinnamic acid, and genistein [30].

In this work we try to value a food widely consumed by the population in Chile and the rest of the world, due to its high nutritional value, rich in fiber, protein, starch, carbohydrates, phenols and low in fat. We consider that the antiplatelet potential of common bean has been little studied, there is only the background of Rodriguez-Arzua et al., 2018, studied landraces of common bean in different stages of growth (green beans, with green pods and green grain; fresh beans, shelled). They evaluated aqueous and methanolic extracts, obtained by maceration and sonication, respectively. Showed that aqueous and methanolic extracts of fresh beans, as well as green beans, showed a similar antiplatelet effect against the agonists ADP and arachidonic acid [31].

In this work, 25 Chilean landraces, very little evaluated to date, were studied. The extraction conditions, ultrasound-assisted extraction technique and microwave-assisted extraction technique were investigated to enhance the protective functionality of this legume. Additionally, we tried to show if there was any relationship between the antioxidant activity evaluated by the ORAC assay and the antiplatelet activity. This allowed us to make a comparison of the extraction methodologies used.

## 2. Results

### 2.1. Extraction Yields of Extracts P. vulgaris L.

The extraction yields in terms of the soluble solid compounds extracted by the microwave and ultrasound-assisted solid-liquid extractions are shown in Table 1.

The soluble solids of the different landraces of Chilean common bean ranged between 0.87 ± 0.06 and 2.27 ± 0.06 Brix. These results indicate the amount of non-water soluble compounds in these extracts. The microwave-assisted extraction showed a higher content of total solids except for the landraces of Blanco Español, Palo, Cabrita, Torcaza, and Cimarron, where the ultrasound extraction showed a higher content of total solids than microwave-assisted extraction. The Cimarron landrace had higher total solids content than the rest of the bean landrace, both for microwave-assisted extraction and for ultrasound-assisted extraction. The Cimarron, Tortola, and Soja extracts obtained by microwave-assisted extraction had twice the total solids content compared to the Bombero extract, this showed the lowest total solids content of 0.87 ± 0.06 Brix.

### 2.2. Total Phenolic Content of Extracts P. vulgaris L.

Previous studies (data not shown) carried out using extraction of compounds with antiplatelet activity using magnetic agitation and orbital shaker showed that the extracts obtained had a lower efficiency in inhibiting platelet aggregation than those extracts obtained by MAE and UAE. It is for this reason that the results obtained with the extracts prepared by MAE and UAE are shown in this work.

Of the 25 landraces of beans, the extracts made by microwave-assisted extraction showed the highest content of phenols in general, highlighting the landraces of Soja and Peumo (893.45 ± 87.30, 866.17 ± 30.28 mg GAE/g, respectively), with the highest contents of total phenols (Table 1). These extracts showed 4 times higher content of total phenols than the microwave-assisted Cisne extract, which showed a lower content of phenols, 220.08 ± 11.98 mg GAE/g shows that the rest of the extracts. The lowest phenol content was obtained in the landraces assisted by ultrasound, except for the landraces of Mantequilla, Lunatus, Cabrita, Pallar Manchado, Blanco Español, and Cisne, where microwave-assisted extraction showed a lower content of total phenols.

### 2.3. Antioxidant Activity of Extracts P. vulgaris L.

In the antioxidant activity using the ORAC test, the extracts made by microwave-assisted extraction also stood out Frutilla (Table 1). The Soja extract obtained by MAE had the highest antioxidant capacity, 35,642.85 ± 2588.88 μmolTE/g, followed by the Peumo and Araucano. These landraces also had the highest total phenol content as we saw earlier. The Lunatus extract using ultrasound-assisted extraction showed the lowest antioxidant capacity, 797.13 ± 158.30 μmolTE/g. Additionally, the extract of Coscorron assisted by ultrasound showed negative ORAC values, the value was below the limit of quantification of the calibration curve. This extract also showed a low content of total phenols, which can be shown in Table 1.

### 2.4. Cytotoxicity of P. vulgaris L. Extracts on Platelets

**Figure 1:**
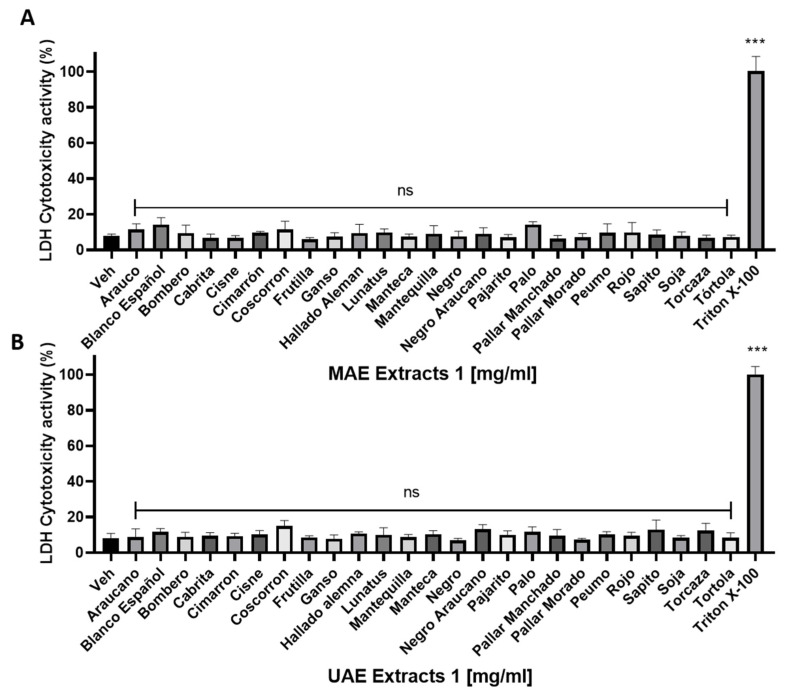
Cytotoxicity on platelets. The results were obtained by LDH release from supernatant on washed platelets and measured at 490 nm in a microplate reader. Data expressed as mean ± SD, *n* = 6 independent trials; Vehicle = PBS. Triton X-100 as a positive control (maximum LDH release). The statistical analysis was performed using the ANOVA (Dunnet test). *** *p* < 0.001 vs. vehicle.

Figure 1 shows the effect of MAE and UAE extracts on LDH release from platelets (cytotoxicity). Values are expressed as mean percentage and SD of 6 independent tests. It is observed 50 extracts (MAE and UAE) do not produce cytotoxicity in washed platelets when compared with the control (vehicle).

### 2.5. Platelet Antiaggregant Activity of Extracts P. vulgaris L.

The antiplatelet screening was carried out with platelet-rich plasma from healthy volunteers where the antiplatelet activity of the 25 landraces of common bean produced by microwave and ultrasound-assisted extraction was evaluated (Table S2). The study of antiplatelet activity showed that bean extracts have greater potential when platelet aggregation is stimulated by TRAP-6 10 µM than by ADP 4 µM. In the case of the latter agonist, the extracts that had the highest percentage of platelet inhibition, around 20%, were the Arauco and Cimarron extracts. While, when platelet aggregation was induced with TRAP-6, significant percentages of platelet aggregation inhibition were observed compared to the control. The extracts made by microwave-assisted extraction showed greater antiplatelet potential than the extracts made by ultrasound-assisted extraction, except for the landraces of Bombero, Cimarron, Hallado Aleman, Lunatus, Palo, Pallar Manchado, and Pallar Morado. These extracts obtained by ultrasound-assisted extraction showed high antiplatelet potential when aggregation was stimulated by TRAP-6 (Table 1).

**Figure 2:**
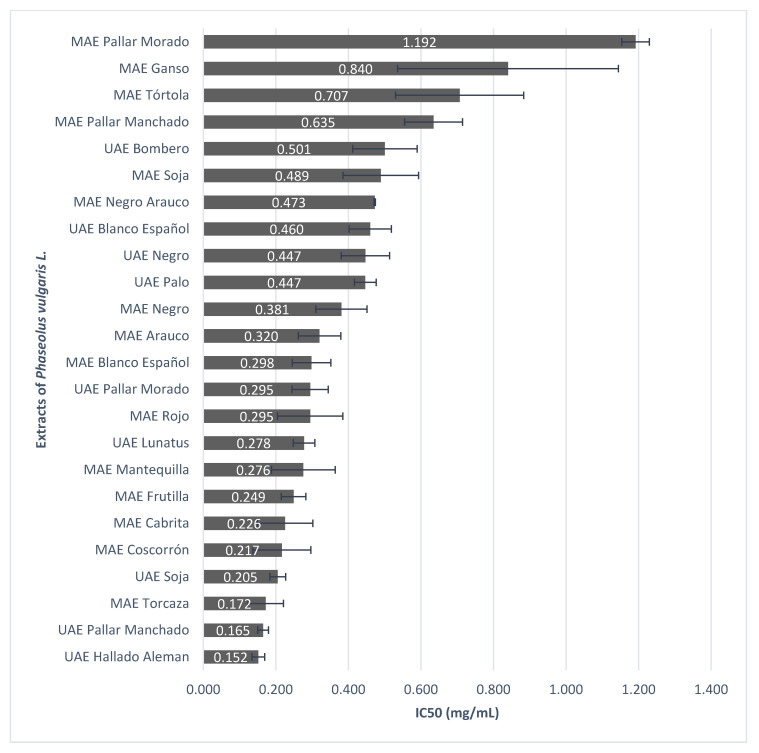
Platelet inhibition of more active extracts of *P. vulgaris* L. The more active extracts, with the percentage of platelet inhibition above 50%, were selected to evaluate the concentration necessary to reduce platelet aggregation by 50% (IC_50_), determined from the concentration curves of the extracts of common bean at 0.025, 0.05, 0.10, 0.25, 0.50, 0.75 and 1.00 mg/mL.

The most active extracts (percentage of platelet inhibition above 50%) obtained by MAE and UAE, from the landraces of common bean, were selected to evaluate the concentration-dependent effect on antiplatelet activity (Figure 2).

24 landraces of beans were studied, of which only 9 extracts were obtained by UAE, the rest by MAE. Although the extracts that showed the greatest inhibition of platelet aggregation were Hallado Aleman (UAE) and Pallar Manchado (UAE), IC_50_ = 0.152 ± 0.018, and 0.165 ± 0.015 mg/mL, respectively.

### 2.6. Total Monomeric Anthocyanin Content of Extracts P. vulgaris L.

Total monomeric anthocyanin contents assays were done to the extracts with more platelet inhibition, these results are show in Table 2.

Based on the results of the total monomeric anthocyanin content analysis performed on bean extracts with the highest platelet inhibition showed in Table 2, it is possible to indicate that the extract with the highest total monomeric anthocyanin content is Pallar Morado followed for Ganso both extracts obtained with MAE extraction process. This higher content of total monomeric anthocyanins could be explained by the presence of spots on the skin of the bean.

## 3. Discussion

In this investigation, extractions by green processes at laboratory scale, MAE and UAE were used. The use of these unconventional techniques provides shorter extraction time, better extraction selectivity, and less thermal decomposition of thermolabile compounds [19,20]. In this study the power used in MAE is low and in UAE the Erlenmeyer flask with the mixture bean/water is submerged in a container with water, which prevents the working temperature from increasing above 50 °C in both methodologies. This would allow avoiding the degradation of thermolabile compounds. Furthermore, they have reduced energy use (mainly due to the speed of the techniques, which implies less energy use), in addition to being technologies that offer high extraction performance, as well as scalable to the industry [18,20,21]. Industrial applications of these processes include the extraction of many natural compounds, such as vitamins, aromas, natural pigments, or essential oils [29,32].

However, there is some evidence that suggest that prolonged exposure to microwaves of high-power levels during MAE may leads to degradation of phenolics compounds (due to their ability to react between them and form smaller or larger compounds as well as form new ones during heating) and the formation of cavitation bubbles during UAE can generates hydroxyl radicals, which can also result in the deterioration of phenolics compounds [18,33,34]. In the methods used, there was a relationship between the selected parameters (mainly time, temperature and solvent), in order to avoid the aforementioned processes.

The results obtained here showed that MAE had better results than UAE. The extracts obtained by the latter generally showed a lower content of total phenols and a lower antioxidant activity. In recent years MAE factors such as potency, solvent, solvent ratio, time, and temperature have been optimized to achieve a high yield of phenolic compounds [18,35].

The MAE process can be relatively selective in the extraction of certain compounds or groups of compounds since dielectric heating depends on the frequency and power of the microwaves [36]. In our study, it was shown that the extraction of phenolic compounds by this technique was carried out efficiently. Most of the common bean landraces studied showed higher ORAC values, highlighting the Soja, Peumo, and Araucano extracts these extracts also had a high content of phenolic compounds. Soja extract had the highest content of total phenols and also the highest antioxidant activity according to the ORAC test.

The results correlated with what was previously reported by Kumar et al., 2019, who reported that the extraction of anthocyanins and phenolic compounds from the seed coat of black Soja bean using MAE produced a maximum of total anthocyanins, 5094.9 mg/L. However, the conventional extraction yield was 1246.89 ± 68.45 mg/L [18,35]. Other authors have revealed that the MAE technique combined with ethanol as a solvent shows a higher yield and extraction quality of green coffee oil [37]. Microwave energy provided localized heating in plant cells, and its driving force could disrupt the plant matrix [38]. It has also been shown that extracts made from cocoa bean shell waste using optimal MAE conditions showed better results than conventional extraction [39]. Other authors have seen that water as a solvent and ultrasound extraction are effective for the extraction of total phenol content (TPC), total flavonoid content (TFC), and antioxidant activity of *Mucuna macrocarpa* (MM) beans [40].

Various assays have been frequently used to estimate antioxidant capacities in fresh fruits and vegetables and their products and foods for clinical studies [41]. The ORAC assay is more relevant because it uses a biologically relevant radical source [42]. Xu et al., 2007 investigated how solvents affect the phenol content and antioxidant capacity of 8 kinds of legumes. The results showed that the polarity of the solvent has significant effects on the total phenolic content, as well as on the rest of the extracted components. The 70% ethanol extracts exhibited the highest ORAC value for all selected legumes [43]. The seed coats of red sword bean (*Canavalia gladiate (Jacq.)* DC.) are rich in antioxidant polyphenols. Under optimal conditions, the antioxidant activity of the extract of this seed obtained by UAE was 755.98 ± 10.23 μmol Trolox/g dry weight, much higher than maceration (558.77 ± 14.42 μmol Trolox/g) or the Soxhlet extraction (479.81 ± 12.75 μmol Trolox/g) [44]. The bean landraces we studied showed higher antioxidant activity than the red sword bean seed coat except for the Coscorrón landrace UAE which could not be detected. The rest of the extracts had higher antioxidant activity according to the ORAC assay.

Except 7 landraces of common bean the rest of the extracts obtained by MAE showed greater antiplatelet potential, stimulated fundamentally with TRAP-6. Extracts of Hallado Aleman (UAE), Pallar Manchado (UAE), and Torcaza (MAE) stood out with higher antiplatelet activity, 0.152 ± 0.018, 0.165 ± 0.015, and 0.172 ± 0.049 mg/mL, respectively. This activity did not correspond to the higher content of phenols or higher antioxidant activity. Hallado Aleman extract had about 1.5 times more phenol content than the Cisne MAE extract (extract with lower content of phenolic compounds). On the other hand, its antioxidant capacity according to the ORAC assay was 14 times higher than the Lunatus extract (lower antioxidant activity by ORAC assay).

There have been few reports showing antiplatelet activity for this legume. Rodriguez et al. [45], showed that the content of total phenols, the content of total flavonoids, the content of total monomeric anthocyanins, and antioxidant capacity in 255 lines of common bean grown under the same environmental conditions have a wide range of variability, with differences always above of an order of magnitude. They showed that bean extracts significantly reduced platelet aggregation induced by adenosine 5′-diphosphate and arachidonic acid.

A large number of metabolites with antiplatelet potential have been identified in common beans (Table 3). An HPLC-MS study of the Black Jamapa bean seed coat revealed the presence of flavonols, anthocyanins, flavanol monomers, and heterogeneous flavanol oligomers. This work reported for the first time in this legume the presence of myricetin glycoside and proanthocyanidin oligomers containing (epi)gallocatechin [46]. Yang et al., 2019, also showed the presence of myricetin, these authors revealed that UAE exhibits a high extraction rate of antioxidants from common bean, showing an extraction efficiency seven times higher than conventional solvent extraction. Some of the compounds detected by this technique were myricetin, cinnamic acid and genistein [30]. Myricetin has been reported to reduce the ability of platelets to spread over collagen and form thrombo in vitro [47]. Genistein suppresses in vitro human platelet aggregation, serotonin secretion, and collagen- and TxA2-induced protein tyrosine phosphorylation. It slightly attenuates thrombin-induced protein tyrosine phosphorylation (100 μg/mL) [47]. Studies suggest that phenolic acid can inhibit platelet adhesion and aggregation [48,49,50,51,52], LDL oxidation [50], protects the heart against oxidative stress [53,54], inhibits platelet inflammatory mediators [50], Inhibit P-selectin expression [49,53] and increases cAMP levels (6,8,9), among others. Along the same lines, flavonoids have also shown the ability to inhibit platelet aggregation induced by various agonists [55,56,57,58], protect against cerebral ischemia and reperfusion injury in rats, enhances cerebrovascular angiogenesis in human umbilical vein endothelial cells [59], inhibit PI3 production [60,61], increases cAMP levels [56] and inhibits levels of inflammatory markers [62]. Anthocyanins have also shown effects similar to those mentioned before [63,64] and, in addition, inhibit P-selectin expression [63,64,65], inhibit the secretion of alpha and dense granules, inhibit PI3K/Akt activation, eNOS phosphorylation and cGMP production [65].

The results of Pearson’s correlation (Figure S1), showed that common bean extracts relationship with phenol content and antioxidant activity (r = 0.68). On the other hand, neither the antioxidant activity nor the phenol content showed a relationship with the antiplatelet activity evaluated in the extracts (r = 0.0037 and r = −0.096, respectively). The antiplatelet activity of numerous fruits and vegetables has been reported to be associated with multiple mechanisms of action [68]. ROS generation has been described as a critical step required for platelet activation, as these species represent important secondary messengers in signal transduction cascades, which may be required for the propagation and activation of platelet aggregation [69]. Sometimes the inhibition of platelet aggregation has been related to the presence of antioxidants. On the other hand, platelets are regulators of multiple processes, with the recruitment of inflammatory cells to sites of injury, release of inflammatory mediators, and regulation of endothelial function [70]. There are many inhibition pathways that have been described for natural products far from a direct effect on ROS, we can mention inhibition of platelet phospholipase C (rutaecarpine, resveratrol and rutin), increased levels of cAMP (allicin, disulfiram, quercetin and guanosine), TXA2 and thrombin receptor antagonists (equol and apigenin), and inositol monophosphate inhibition (marchantinquinone) [71].

It has been shown that the difference between the antiplatelet potential can be attributed to the individual action of each secondary metabolite and can be enhanced from their interactions through a supposed synergy or cooperativity between the metabolites [8]. However, it has been shown that there are many factors that influence the composition of an extract and therefore its biological potential, among these variables we can mention genotype, solvent, extraction method, environment, processing, storage and harvest stage [8,72]. A study carried out on the berries showed that the antiplatelet activity varies according to the genotype, the solvent used and the organ of the plant, as well as the state of maturity of the fruit. Rodriguez et al., 2018. revealed that the immature fruit had a high antiplatelet potential, which was related to its high phenol content and lower anthocyanin content, unlike the mature fruit, which was richer in anthocyanins and has been described as having a high antioxidant potential [72,73]. These results are evidence that antioxidant activity is sometimes not related to a high antiplatelet potential. The same was observed in this study, where it was shown that extracts with higher phenol content and higher ORAC value (antioxidant capacity) did not have greater antiplatelet potential. In other works, we will study the chemical profile of the extracts with the highest antioxidant and antiplatelet activity, in order to show the differences in the chemical composition of each exact, which should be related to the findings of antiplatelet inhibition found for the extracts of common bean.

## 4. Materials and Methods

### 4.1. Plant Material

Samples of the 24 different Chilean landraces *Phaseolus vulgaris* L. were obtained from small producers in the south-central macrozone of Chile (Table 4), all bean samples corresponded to landraces from the year 2021, Bean cultivars are mainly composed of protein, dietary fiber, minerals, and vitamins [74].

Once the samples were obtained, they were ground using a domestic juicer, and subsequently sieved with a 450-micron sieve, mesh 40. This grinding was carried out to reduce the size of the particles, to increase the effective contact area between the particle and water, used as extraction solvent as is described for Panja [23], and for Llompart and coworkers [75]. The above procedure then allows increasing the extraction yields of target compounds.

### 4.2. Preparation of Extracts

To obtain the extracts, about 1 g of each of the 25 previously ground bean samples was used and put in contact with 40 milliliters of distilled water in an Erlenmeyer flask. Thus, extraction assays were developed using a ratio of beans/water of 1/40, for one hour. This relationship was selected to not saturate the extraction solvent, and thus increase the extraction yields. Microwave-assisted solid-liquid extractions were carried out under the next operational variables:

- Ultrasound-assisted extraction (UAE): An ultrasound bar (QSonica Q125, Newton, CT, USA) was used, at 50% amplitude (10 kHz) with a sonication time of 60 min, to prevent the temperature from rising too high, the Erlenmeyer flask with the mixture of beans and distilled water was placed inside of an ice bath.
- Microwave-assisted extraction (MAE): A 1200 W domestic microwave (WMW606ADWC Whirlpool, Shanghai, China) was used. To carry out the microwave-assisted extraction, the bean mixtures with distilled water in an Erlenmeyer flask, were placed inside them in a 1/40 ratio. The minimum power of 1 was fixed, which is equivalent to 120 W. The extraction time was 60 min.

The samples obtained in the microwave and ultrasound-assisted extraction processes were centrifuged 50 mL conical tubes in a centrifuge (K2015R Centurion Scientific, Manchester, UK) at 3500 rpm for 15 min at 20 °C. Subsequently, the supernatant was separated with Corning Falcon cell filters of 100, 70, and 40 µm respectively, to obtain the supernatant, which was frozen at −86 °C for 48 h, then freeze-dried (Operon, FDU 7024, Gimpo, Korea) for 20 h with a cold trap (−70 °C) and stored until analysis.

### 4.3. Total Soluble Solids and Total Phenolics

For the determination of soluble solids, a digital refractometer (Hanna Instruments HI 96801, Woonsocket, RI, USA) was used. On the calibrated refractometer and at room temperature (20 °C), 0.5 mL of filtered supernatant was deposited, and the result was read and expressed as °Brix, which is equivalent to the weight/weight percentage. The reading procedure was carried out in triplicate.

On the other hand, total phenol content was determined by the Folin-Ciocalteu method. This method quantifies the reducing power of phenolic compounds on the Folin-Ciocalteu reagent, through the formation of a blue complex that is read at 750 nm [76]. The methodology used is based on OIV-MA-AS2-10, Compendium of International Methods of Analysis—OIV Folin-Ciocalteu Index, 2019 edition. Briefly, a calibration curve was made with different concentrations from a primary solution of 5 g/L of gallic acid. For the reading of the samples, the following procedure was followed. In a 10 mL flask, the reagents were added in the following order: 250 µL of sample, standard or water in the case of the blank; 5 mL of MilliQ water; 500 µL of Folin’s reagent; 2 mL of sodium carbonate p/v solution. It was made up to volume with MiliQ water, stirred and subsequently stored for 30 min in the dark at room temperature. Finally, absorbance was measured in a spectrophotometer (Spectro UV-11, MCR, Shanghai, China) at 750 nm.

### 4.4. Total Monomeric Anthocyanin Content

Determination of total monomeric anthocyanin content was determined according to the official method of the AOAC [77]. This method is based on the measurement of the color change of monomeric anthocyanins pigments, which change color reversibly with a change in pH; the colored form of oxonium exists at pH 1.0, and the colorless hemiketal form predominates at pH 4.5. The difference in the absorbance of the pigments at 520 nm is proportional to the pigment concentration. The results are expressed in a cyanidin-3-glucoside base. It should be noted that anthocyanins degraded in polymeric forms are not included in the measurements because they absorb at pH 4.5 as well as pH 1.0, determine the appropriate dilution factor by diluting the sample with pH 1 buffer, until absorbance at 520 is within the linear range of the spectrophotometer.

To carry out this measurement, a pH 1 buffer of potassium chloride (0.025 M) and a pH 4.5 buffer of sodium acetate (0.4 M) were used. Two dilutions of the samples to be analyzed were prepared, one with the pH 1 buffer and the other using the pH 4.5 buffer, subsequently the absorbance at 520 and 700 nm was determined against a blank cell, which was filled with distilled water.

Total monomeric anthocyanins were calculated using the following equation:

$$Total\;monomeric\;Anthocyanin\;(cyanidin-3-glucoside\;equivalents,\;mg/L=\frac{A*\;MW*DF\;x10_{}^{3}}{Ɛ*I}$$

where *A* = (*A*_520nm_ − *A*_700nm_) pH 1.0 − (*A*_520nm_ − *A*_700nm_) pH 4.5; *MW* (Molecular Weight) = 449.2 g mol^−1^ for cyanidin-3-glucoside; *DF* = Dilution Factor; l = pathlength in cm; Ɛ = 26 900 molar extinction coefficient, in L × mol^−1^ cm^−1^, for cyanidin-3-glucoside; and 10^3^ = factor for conversion from g to mg.

### 4.5. Antioxidant Activity (ORAC)

There are various methodologies to measure the scavenging capacity of free radicals, such as ORAC, ABTS, DPPH, DMPD, among others. ORAC (Oxygen Radical Absorbance Capacity) is the analytical method most implemented for antioxidant analysis due to its adaptability to the great need of radical species and conditions of various laboratories. In addition to being done under conditions like those physiological and with the superoxide anion which is a radical with high biological relevance [78].

For the analysis of antioxidant capacity by ORAC, the metholody described for Ou and coworkers [79] was used a fluorescence microplate reader with an excitation filter at 485 ± 20 nm and an emission filter at 538 ± 25 nm (Synergy HTX Multi-Mode Reader, Biotek, Santa Clara, CA, USA) was used. This instrument is capable of evaluating UV-Vis absorbance, fluorescence, and luminescence. Briefly, sodium fluorescein (0.015 mg mL^−1^), AAPH radical solution (120 mg mL^−1^), and Trolox standard solution (100 µM) were prepared with phosphate buffer (75 mM, pH 7). The operating conditions for the final reaction consisted of 50 μL of diluted extract, Trolox standard or phosphate buffer (blank), 50 μL of fluorescein, and 25 μL of AAPH incubated at 37 °C in the microplate reader. For the measurement, black plates with 96 wells were used allowing the analysis of three samples per plate. Gallic acid was used as a control. The fluorescence was recorded every 5 min over 60 min using the KC4 TM software (BioTek Instruments, Winooski, VT, USA), differences of areas under the fluorescence decay curve (AUC) between the blank and the sample over time were compared and the results were expressed as μM Trolox Equivalents

### 4.6. Preparation of Human Platelets

Whole blood was obtained by venous phlebotomy from healthy volunteer donors (7 days without NSAID use) who accepted informed consent, as previously described [80]. The study protocol was approved by the Scientific Ethics Committee from Universidad de Talca (Protocol N° 29/2021). Whole blood was centrifuged at 240× *g* to obtain platelet-rich plasma (PRP) as previously described [8]. Subsequently, the platelet count was adjusted to 300 × 10^9^ platelet/L with platelet-poor plasma (PPP) obtained by centrifugation at 800× *g*.

### 4.7. Obtaining Washed Platelets

Whole blood was anticoagulated with acid-citrate-dextrose (ACD) (*v*/*v* ratio 4:1) and centrifuged for 12 min at 240× *g* to obtain platelet-rich plasma (PRP). The PRP was centrifuged for 8 min at 900× *g* in a microcentrifuge. The pellet (platelets) was resuspended in Buffer Tyrodes without calcium: ACD (*v*/*v* ratio 5:1) and centrifuged again for 8 min at 900× *g*. Finally, the platelets were resuspended in Buffer Tyrodes without calcium and adjusted to the desired count by reading the hematology counter (Mindray BC-3000 Plus, Shenzhen, China) [81].

### 4.8. Release of Lactate Dehydrogenase (LDH)

To assess the cytotoxicity, the LDH Cytotoxicity Assay Kit (Cayman Chemical, Ann Arbor, MI, USA) was used. Washed platelets (200 × 10^9^ platelets/L) were incubated with the highest concentration of extracts tested (1 mg/mL) or vehicle for 10 min at 37 °C. Subsequently, the platelet solution was centrifuged at 900× *g* for 8 min in a microcentrifuge to obtain the cell supernatant, which was reacted with the kit’s working reagent. As a positive control of 100% cytotoxicity (maximum LDH release), a 10% Triton X-100 solution was used on washed platelets [82].

### 4.9. Platelet Aggregation

The antiplatelet activity of the beans obtained by green processes was performed by turbidimetry using a lumi-agregometer as previously described [9]. The PRP was incubated with the different extracts at a concentration of 1 mg/mL for 4 min. Platelet aggregation was induced with the agonists ADP 4 µM and TRAP-6 10 µM, these agonists act on different platelet receptors [80]. Additionally, PRP was incubated with PBS as a positive control of platelet aggregation. The percentage of platelet aggregation was obtained with the AGGRO/LINK software (Chrono-Log, Havertown, PA, EE. UU.). The results were expressed as inhibition of platelet aggregation. Once the antiplatelet activity was evaluated at 1 mg/mL, the concentration-dependent antiplatelet potential was studied. The extracts that inhibited platelet aggregation by more than 50% were selected and the concentration necessary to reduce platelet aggregation by 50% (IC_50_) was determined from the concentration curves of the extracts of common bean (0.025, 0.05, 0.10, 0.25, 0.50, 0.75, 1.00 mg/mL).

### 4.10. Statistical Analysis

Data were analyzed using Prism 8.0 software (GraphPad Inc., San Diego, CA, USA) and expressed as mean ± standard deviation (SD). Considering the small size of the experiments to determine the normality of the data, the Shapiro-Wilk test was used. For the analysis of the platelet aggregation results, a one-way analysis of variance (ANOVA) was used to detect if there were statistical differences between the groups and the Dunnet’s postdoc test to make a comparison with the control (vehicle) [80]. While Tuckey’s was used to compare the differences between various groups, 25 landraces [83]. The differences between groups were analyzed using a one-way analysis of variance (ANOVA) and Tukey’s posthoc test. The Pearson correlation coefficient was used to determine the differences between the conditions and variables studied. *p* values <0.05 were considered statistically significant (Figure S1) [8].

## 5. Conclusions

This study evidence extraction efficiency between the green processes, MAE and UAE, to obtain extracts of 25 landraces of common bean with antioxidant and antiplatelet properties. Our studies reveal that the antiplatelet potential of bean extracts is not related to the presence of antioxidants (ROS inhibition pathway). Other studies are needed to help us elucidate the eventual mechanism by which the most active bean extracts inhibit platelet aggregation considering the chemical profile of the extracts and the different pathways mentioned above.

## Figures and Tables

**Table 1:** Results of the content of soluble solids, total phenols and antioxidant activity of 25 landraces of *P. vulgaris* L.

Landraces	Extraction Technique	Soluble Solid Content	TPC (mg GAE/g)	ORAC μmolTE/g	TRAP-6 10 µM PA (%)	ADP 4 µM PA (%)
Arauco	MAE	1.50 ± 0.00 ^a^	572.58 ± 10.21 ^a^	22,725.36 ± 691.51 ^a^	26.8 ± 1.2 ^a^	71.3 ± 1.5 ^a^
	UAE	1.33 ± 0.06 ^a^	464.94 ± 10.36 ^a^	9048.36 ± 289.97 ^b^	49.1 ± 3.5 ^b^	71.0 ± 2.5 ^a^
Blanco Español	MAE	0.93 ± 0.06 ^b^	241.94 ± 18.17 ^b^	4709.13 ± 1233.93 ^c^	23.8 ± 2.6 ^a^	75.7 ± 2.2 ^ab^
	UAE	0.97 ± 0.06 ^b^	280.27 ± 46.02 ^b^	2721.33 ± 257.49 ^c^	36.2 ± 3.0 ^ab^	71.5 ± 3.0 ^a^
Bombero	MAE	1.00 ± 0.17 ^b^	311.73 ± 4.42 ^ba^	3775.75 ± 966.34 ^c^	77.5 ± 3.1 ^c^	92.3 ± 0.8 ^cb^
	UAE	0.90 ± 0.06 ^b^	227 ± 22.95 ^b^	2821.35 ± 70.83 ^c^	17.8 ± 2.4 ^a^	71.5 ± 2.4 ^a^
Cabrita	MAE	1.00 ± 0.10 ^b^	264.66 ± 11.62 ^ba^	8045.22 ± 600.05 ^b^	36.3 ± 3.1 ^ab^	84.7 ± 0.8 ^b^
	UAE	1.27 ± 0.06 ^ab^	437.89 ± 36.74 ^a^	14,489.49 ± 344.15 ^db^	65.0 ± 3.2 ^cb^	87.9 ± 1.4 ^b^
Cimarrón	MAE	2.03 ± 0.06 ^c^	520.47 ± 31.28 ^a^	2722.64 ± 932.78 ^c^	79.1 ± 3.4 ^c^	73.8 ± 3.8 ^a^
	UAE	2.27 ± 0.06 ^c^	352.27 ± 34.03 ^ba^	9612.22 ± 534.08 ^b^	78.6 ± 2.4 ^c^	84.2 ± 1.3 ^b^
Cisne	MAE	1.07 ± 0.06 ^ab^	220.08 ± 11.98 ^b^	3657.96 ± 386.98 ^c^	60.1 ± 4.1 ^cb^	80.8 ± 2.5 ^ba^
	UAE	0.87 ± 0.06 ^b^	246.85 ± 17.66 ^ba^	3310.31 ± 301.25 ^c^	72.4 ± 3.7 ^c^	86.5 ± 1.2 ^b^
Coscorrón	MAE	1.27 ± 0.06 ^ab^	280.06 ± 38.53 ^ba^	2704.07 ± 482.92 ^c^	7.9 ± 0.4 ^d^	73.8 ± 1.9 ^a^
	UAE	0.97 ± 0.06 ^b^	225.19 ± 11.30 ^ba^	Nd	54.9 ± 1.0 ^b^	84.8 ± 0.8 ^b^
Frutilla	MAE	1.10 ± 0.00 ^ba^	414.45 ± 29.54 ^a^	21,630.05 ± 260.90 ^a^	21.3 ± 1.1 ^a^	82.1 ± 1.8 ^b^
	UAE	1.07 ± 0.06 ^b^	383.32 ± 39.47 ^ba^	14,238.93 ± 910.50 ^db^	50.3 ± 1.7 ^b^	85.4 ± 0.6 ^b^
Ganso	MAE	1.70 ± 0.10 ^a^	431.83 ± 34.86 ^a^	9375.30 ± 1722.49 ^b^	17.1 ± 2.7 ^a^	74.1 ± 1.2 ^a^
	UAE	1.47 ± 0.06 ^a^	318.96 ± 27.29 ^ba^	3313.60 ± 351.80 ^c^	69.8 ± 3.9 ^c^	82.3 ± 0.7 ^b^
Hallado Alemán	MAE	1.00 ± 0.00 ^b^	541.85 ± 33.37 ^a^	17,682.97 ± 248.60 ^d^	85.8 ± 0.7 ^c^	81.8 ± 1.2 ^b^
	UAE	0.93 ± 0.15 ^b^	337.91 ± 19.47 ^ba^	11,321.46 ± 987.80 ^bd^	11.0 ± 0.9 ^ad^	73.3 ± 2.9 ^a^
Lunatus	MAE	1.53 ± 0.06 ^a^	283.09 ± 69.11 ^ba^	5857.60 ± 2350.40 ^b^	86.8 ± 0.8 ^c^	90.8 ± 0.4 ^cb^
	UAE	1.47 ± 0.12 ^a^	298.27 ± 26.91 ^ba^	797.13 ± 158.30 ^c^	7.8 ± 1.4 ^d^	72.6 ± 1.6 ^a^
Manteca	MAE	1.53 ± 0.06 ^a^	603.89 ± 6.83 ^ac^	12,561.54 ± 1096.83 ^bd^	55.2 ± 1.3 ^b^	79.8 ± 1.4 ^ab^
	UAE	1.00 ± 0.00 ^b^	321.96 ± 5.92 ^ba^	2867.47 ± 214.95 ^c^	78.5 ± 2.9 ^c^	87.9 ± 1.2 ^bc^
Mantequilla	MAE	1.50 ± 0.00 ^a^	308.89 ± 9.40 ^ba^	13,721.58 ± 3113.94 ^db^	22.3 ± 1.8 ^a^	76.7 ± 2.4 ^ab^
	UAE	0.93 ± 0.06 ^b^	340.85 ± 22.87 ^ba^	3976.00 ± 463.28 ^c^	56.3 ± 1.3 ^b^	76.4 ± 1.8 ^ab^
Negro	MAE	1.30 ± 0.00 ^a^	806.90 ± 6.66 ^c^	16,659.61 ± 158.45 ^d^	7.8 ± 1.2 ^d^	73.6 ± 2.0 ^a^
	UAE	1.00 ± 0.10 ^b^	543.40 ± 12.02 ^a^	7243.07 ± 964.23 ^b^	25.3 ± 1.4 ^a^	84.0 ± 0.9 ^bc^
Negro Arauco	MAE	1.27 ± 0.02 ^a^	345.89 ± 47.58 ^ba^	8955.42 ± 516.75 ^b^	28.5 ± 2.8 ^a^	72.8 ± 2.0 ^a^
	UAE	1.17 ± 0.02 ^ba^	323.62 ± 77.94 ^ba^	3481.76 ± 759.27 ^c^	47.0 ± 1.7 ^b^	78.0 ± 1.4 ^ab^
Pajarito	MAE	1.33 ± 0.06 ^a^	594.36 ± 8.80 ^a^	18,723.60 ± 1700.55 ^ad^	56.7 ± 0.9 ^b^	84.1 ± 1.1 ^bc^
	UAE	1.23 ± 0.06 ^a^	485.70 ± 18.84 ^a^	12,904.29 ± 968.70 ^bd^	78.0 ± 2.8 ^c^	87.7 ± 1.3 ^bc^
Palo	MAE	1.30 ± 0.10 ^a^	423.06 ± 27.19 ^a^	17,828.49 ± 826.24 ^d^	85.4 ± 0.3 ^c^	81.0 ± 3.3 ^bc^
	UAE	1.33 ± 0.06 ^a^	391.23 ± 50.86 ^ba^	16,179.95 ± 2142.43 ^d^	23.5 ± 1.5 ^a^	71.1 ± 3.4 ^a^
Pallar Manchado	MAE	0.93 ± 0.06 ^b^	316.52 ± 93.50 ^ba^	15,983.88 ± 674.45 ^d^	20.9 ± 1.0 ^a^	88.0 ± 1.3 ^bc^
	UAE	0.90 ± 0.00 ^b^	374.63 ± 53.69 ^ba^	9331.82 ± 891.31 ^b^	18.7 ± 1.1 ^a^	74.0 ± 0.6 ^a^
Pallar Morado	MAE	1.17 ± 0.06 ^ab^	795.95 ± 29.97 ^c^	11,233.61 ± 1702.32 ^b^	31.9 ± 0.6 ^ab^	73.5 ± 2.6 ^a^
	UAE	1.03 ± 0.06 ^b^	495.63 ± 39.09 ^a^	10,993.16 ± 476.40 ^b^	20.8 ± 1.0 ^a^	68.0 ± 1.6 ^a^
Peumo	MAE	1.40 ± 0.10 ^a^	866.17 ± 30.28 ^c^	25,642.98 ± 2396.79 ^a^	46.1 ± 6.2 ^b^	81.8 ± 1.9 ^b^
	UAE	0.90 ± 0.00 ^b^	720.52 ± 18.43 ^c^	22,596.47 ± 3452.67 ^a^	81.7 ± 1.7 ^c^	82.9 ± 0.5 ^b^
Rojo	MAE	1.00 ± 0.00 ^b^	590.86 ± 24.25 ^a^	12,809.11 ± 976.94 ^b^	35.5 ± 2.7 ^ab^	75.0 ± 2.0 ^ba^
	UAE	0.90 ± 0.10 ^b^	518.22 ± 11.90 ^a^	6535.65 ± 1872.82 ^b^	57.1 ± 7.5 ^bc^	69.0 ± 7.0 ^a^
Sapito	MAE	1.17 ± 0.06 ^ab^	265.30 ± 12.93 ^ba^	5184.58 ± 359.74 ^b^	69.6 ± 4.7 ^c^	69.0 ± 4.8 ^a^
	UAE	1.13 ± 0.06 ^ab^	232.68 ± 4.14 ^ba^	4345.33 ± 26.73 ^c^	79.4 ± 3.0 ^c^	87.8 ± 0.9 ^bc^
Soja	MAE	1.83 ± 0.06 ^a^	893.45 ± 87.30 ^c^	35,642.85 ± 2588.88 ^e^	22.4 ± 2.8 ^a^	75.2 ± 2.2 ^ab^
	UAE	1.63 ± 0.06 ^a^	696.85 ± 21.06 ^ca^	19,820.36 ± 1474.80 ^a^	28.1 ± 2.9 ^a^	74.0 ± 1.9 ^a^
Torcaza	MAE	1.57 ± 0.06 ^a^	349.19 ± 16.56 ^ab^	5720.66 ± 145.28 ^b^	27.5 ± 2.0 ^a^	78.7 ± 3.4 ^ab^
	UAE	1.63 ± 0.12 ^a^	302.92 ± 15.42 ^ba^	3337.06 ± 888.64 ^c^	48.0 ± 0.7 ^b^	66.2 ± 3.9 ^a^
Tórtola	MAE	1.90 ± 0.00 ^c^	327.65 ± 24.89 ^ba^	13,617.59 ± 1519.75 ^bd^	12.0 ± 1.3 ^da^	78.2 ± 1.7 ^ab^
	UAE	1.00 ± 0.10 ^b^	284.80 ± 6.42 ^ba^	3561.15 ± 880.60 ^c^	75.06 ± 2.9 ^c^	80.8 ± 1.2 ^b^

Data are expressed as mean ± SD, *n* = 3 from at least three independent experiments. Nd: unidentified (value was below the limit of quantification of the calibration curve). Different superscript letters (a–e) in the same column show significant differences within each landrace, according to Tukey’s test (*p* < 0.05).

**Table 2:** Total monomeric anthocyanin on extracts.

Extracts	Cyanidin-3-glucoside (mg/100 g of Sample)
MAE Pallar Morado	25.81 ± 0.02
MAE Ganso	24.86 ± 0.04
UAE Bombero	23.58 ± 0.03
MAE Tortola	12.72 ± 0.02
MAE Soja	12.33 ± 0.03
UAE Palo	10.58 ± 0.01
MAE Pallar Manchado	9.83 ± 0.01
MAE Negro Arauco	9.27 ± 0.01
UAE negro	4.42 ± 0.01
UAE Blanco Español	0.86 ± 0.00

**Table 3:** Compounds identified on *P. vulgaris* L. with cardioprotective potential.

Bioactive Compound	Cardioprotective Effect and/or Mechanisms	Reference	
Phenolic acids (coumaric, chlorogenic, ferulic, cinnamic, sinapic, protocatechuic, vanillic, syringic acid)	Inhibit platelet adhesion and/or aggregation	[48,49,50,51,52]	
	Inhibit LDL oxidation	[66]	
	Protects the heart against oxidative stress	[53,54]	
	Inhibits platelet inflammatory mediators	[50]	
	Increases cAMP levels	[50,51,52]	
	Inhibit P-selectin expression	[49,53]	
	activation of cAMP and cGMP signaling pathways	[50,51,52]	
Flavonoids (Myricerin, Genistein, Glycitein, Formononetin, Naringenin, Hesperetin, Daidzein, Catechin, Kaemferol-3-glucoside, Taxifolin, Apigenin-7 Glycoside, Luteolin-7 Glycoside)	Inhibit platelet aggregation induced by several agonists	[55,56,57,58]	
	Inhibit PI3 production	[59,60,61]	
	Protect against cerebral ischemia and reperfusion injury in rats and enhances cerebrovascular angiogenesis in human umbilical vein endothelial cells	[59]	
	Inhibits levels of inflammatory markers	[62]	
	Increases cAMP levels	[56]	
Anthocyanins (Delphinidin, Petunidin, Malvidin, Cyanidin-3-O-glucoside, Pelargonidin-3-O-glucoside)	Inhibit platelet aggregation induced by several agonists	[63,67]	
	Inhibit the secretion of alpha and dense granules	[65]	
	Inhibit PI3K/Akt activation, eNOS phosphorylation and cGMP production	[63]	
	Inhibit P-selectin expression	[63,64,65]	

Reprinted with permission from ref. [13]. Rodríguez, L., et al., 2022.

**Table 4:** Description of the landraces of *P. vulgaris* L.

Chilean *Phaseolus vulgaris* L. Landraces	Country	Region	Village	GPS Coordinate
Bombero, Cimarrón, Cisne, Ganso, Tórtola, Lunatus	Chile	Maule	Curepto, Maule	−34°.99′09″34, −72°.01′56″31
Arauco, Palo, Pallar Manchado, Pallar Morado, Negro Arauco, Hallado Alemán, Manteca	Chile	Maule	Hualañe	−35°.01′44″19, −71°.73′94″57
Cabrita, Coscorrón, Pajarito, Frutilla, Peumo, Rojo, Sapito	Chile	Ñuble	Chanco, Cobquecura	−36°.28′66″29, −72°.71′92″36
Blanco Español, Torcaza, Negro, Mantequilla	Chile	Maule	Longaví	−35°.96′80″49, −71°.71′36″48

## Data Availability

Not applicable.

## Supplementary Materials

The following supporting information can be downloaded at: https://www.mdpi.com/article/10.3390/plants11091179/s1, Table S1. Content of bioactive compounds for different varieties and extraction technique. Table S2. Antiplatelet activity of 25 varieties of *P. vulgaris* L. Figure S1. Correlation between phenol content, antioxidant activity, and antiplatelet activity of extracts *P. vulgaris* L. References [84,85,86,87] are cited in the supplementary materials.
