# Supplementary material for: Antioxidant Capacity and Antiplatelet Activity of Aqueous Extracts of Common Bean (Phaseolus vulgaris L.) Obtained with Microwave and Ultrasound Assisted Extraction
Source: Plants (Basel). 2022 Apr 27;11(9):1179. doi: 10.3390/plants11091179 (PMC9102907; doi:10.3390/plants11091179)
Supplement: Supplementary file 1 [file plants-11-01179-s001.zip › plants-1663404-supplementary.pdf]

Supporting Information

Table S1. Content of bioactive compounds for different varieties and extraction technique.

Bioactive compound	Content (reference)	Variety	Extraction technique
**Total phenolic**	1364.5 mg GAE/g dw [1]	rojo	CSE
	114.9 mg GAE/g dw [1]	blanco	CSE
	1043.3 mg GAE/g dw [1]	pinto colorado	CSE
	550.1 to 787.5 mg GAE/g dw [1]	negro (different varietys)	CSE
	451.9 mg GAE/g dw [1]	barreton	CSE
	15.5 mg GAE/g coat [2]	negro	CSE
	5.46 mg GAE/g coat [2]	Pinto Saltillo	CSE
	4.69 mg GAE/g dw/ 29.37 mg GAE/g coat [3]	Negro	MAE
	5.77 mg GAE/g dw/ 39.98 mg GAE/g coat [3]	Small Red	MAE
	1.86 mg GAE/g d/ 14.69 mg GAE/g coat [3]	Negro	CSE
	1.74 mg GAE/g dw/ 20.35 mg GAE/g coat [3]	Small Red	CSE
	352.14 to 4664.92 mg GAE/100 mL [4]	Black Soybean	MAE
	4871 mg GAE/100 g DW [5]	Pinto Kidney	CSE
	165.524 μg GAE/gr (DW) [6]	Volturara	CSE
	997.774 μg GAE/gr (DW) [6]	Zampognaro	CSE
	1150.474 μg GAE/gr (DW) [6]	Screziato impalato	CSE
	1291.62 μg GAE/gr (DW) [6]	Nero di Caposele	CSE
	20.49 to 31.31 mg GAE/g [7]	Mung	UAE

	0.59 to 29.58 mg GAE/g [8]	Mung	CSE
	0.40 to 54.45 mg GAE/g [8]	Adzuki	CSE
**Flavonoid**	19.9 mg QE/g dw [1]	rojo	CSE
	1.1 mg QE/g dw [1]	blanco	CSE
	5.7 mg QE/g dw [1]	pinto colorado	CSE
	5.6 to 18.6 mg QE/g dw [1]	negro (different varietys)	CSE
	1.2 mg QE/g dw [1]	barreton	CSE
	0.083 mg RUE/g coat [2]	Pinto Saltillo	CSE
	1116.94 to 12310.78 mg QE/L [4]	Black Soybean	MAE
	4477 mg CE/100 g DW [5]	Pinto	CSE
	3529 mg CE/100 g DW [5]	small speckled kidney	CSE
	51.511 µg (QE)/gr (DW) [6]	Volturara	CSE
	562378 µg (QE)/gr (DW) [6]	Zampognaro	CSE
	703.336 µg (QE)/gr (DW) [6]	Screziato impalato	CSE
	542.337 µg (QE)/gr (DW) [6]	Nero di Caposele	CSE
	0.39 to 0.71 mg CE/g [6]	Mung	UAE
	0.40 to 22.08 mg CE/g [6]	Mung	CSE
	0.38 to 50.78 mg CE/g [6]	Adzuki	CSE
**Anthocyanin**	0.59 mg cyaniding 3-glucoside/g seed coat [1]	rojo	CSE
	0.69 mg cyaniding 3-glucoside/g seed coat [1]	pinto colorado	CSE
	2.82 to 9.42 mg cyaniding 3-glucoside/g seed coat [1]	negro (different varietys)	CSE

	0.10 mg cyaniding 3-glucoside/g seed coat [1]	barreton	CSE
	206.0 to 250.0 mg/100 g coat [2]	negro (different varietys)	CSE
	3.0 mg/100 g coat [2]	FM-Eugenia	CSE
	876.68 to 5891.23 mg/L [4]	Black Soybean	MAE
	5.37 mg CE/g DW [5]	Kidney	CSE
	28.0 to 158 mg CE/g DW [5]	Pinto	CSE
	10.706 μg (C3GE)/gr (DW) [6]	Zampognaro	CSE
	7.255 μg (C3GE)/gr (DW) [6]	Screziato impalato	CSE
	63.278 μg (C3GE)/gr (DW) [6]	Nero di Caposele	CSE
**Tanin**	3.40 mg CAE/g dw [1]	rojo	CSE
	0.53 mg CAE/g dw [1]	blanco	CSE
	1.42 mg CAE/g dw [1]	pinto colorado	CSE
	1.62 to 5.57 mg CAE/g dw [1]	negro (different varietys)	CSE
	0.80 mg CAE/g dw [1]	barreton	CSE
	60.6 mg GAE/g coat [2]	Pinto Saltillo	CSE
	6.81 to 52.64 mg CE/g [8]	Mung	CSE
	3.59 to 97.42 mg CE/g [8]	Adzuki	CSE

Table S2. Antiplatelet activity of 25 varieties of *P. vulgaris* L.

Extracts		TRAP-6 10μM			ADP 4μM		
		PA (%)	AUC	Slope	PA (%)	AUC	Slope
**Arauco**	**MAE**	26,8 ± 1,2 ***	41,8 ± 3,4	47,8 ± 2,0	71,3 ± 1,5 *	286 ± 10,6	82,6 ± 1,4
	**UAE**	49,1 ± 3,5 ***	296,2 ± 1,4	27,1 ± 3,4	71 ± 2,5 **	295,4 ± 1,1	81,2 ± 6,8
**Blanco Español**	**MAE**	23,8 ± 2,6 ***	133,4 ± 28	53,8 ± 5,4	75,7 ± 2,2 ns	295,2 ± 1,1	108,2 ± 3,9
	**UAE**	36,2 ± 3,0 ***	182,4 ± 28,8	46,8 ± 6,3	71,5 ± 3,0 **	295,6 ± 0,8	66,5 ± 7,4
**Bombero**	**MAE**	77,5 ± 3,1 ns	284,2 ± 2,7	100,8 ± 6,9	92,3 ± 0,8 ns	309,8 ± 5,1	75,2 ± 5,1
	**UAE**	17,8 ± 2,4 ***	128,8 ± 19,2	52,2 ± 3,9	71,5 ± 2,4 **	286,4 ± 16,7	62,7 ± 4,5
**Cabrita**	**MAE**	36,3 ± 3,1 ***	297,4 ± 0,5	13,4 ± 2,2	84,7 ± 0,8 ns	295,6 ± 0,9	78,4 ± 6,6
	**UAE**	65 ± 3,2 *	292 ± 1,8	39,1 ± 3,6	87,9 ± 1,4 ns	299,6 ± 0,1	75,8 ± 1,0
**Cimarrón**	**MAE**	79,1 ± 3,4 ns	314,2 ± 15	83,4 ± 8,0	73,8 ± 3,8 *	270 ± 7,4	77,9 ± 6,8
	**UAE**	78,6 ± 2,4 ns	278,2 ± 9,4	57,1 ± 10,9	84,2 ± 1,3 ns	298,2 ± 0,8	55,3 ± 1,8
**Cisne**	**MAE**	60,1 ± 4,1 **	289,4 ± 3,4	77,4 ± 2,0	80,8 ± 2,5 ns	294,8 ± 0,1	506,7 ± 114,2
	**UAE**	72,4 ± 3,7 ns	291 ± 1,1	32,9 ± 4,5	86,5 ± 1,2 ns	296,8 ± 0,3	53,9 ± 1,5

**Coscorrón**	**MAE**	7,9 ± 0,4 ***	83,6 ± 23,3	31,6 ± 1,4	73,8 ± 1,9 *	222 ± 21,4	106,9 ± 12,2
	**UAE**	54,9 ± 1,0 ***	292,6 ± 1,4	46,9 ± 12,1	84,8 ± 0,8 ns	296,2 ± 1,1	58 ± 1,9
**Frutilla**	**MAE**	21,3 ± 1,1 ***	134,8 ± 28,8	47,5 ± 4,3	82,1 ± 1,8 ns	298,4 ± 0,1	52 ± 6,3
	**UAE**	50,3 ± 1,7 ***	287,4 ± 1,6	48,5 ± 2,7	85,4 ± 0,6 ns	295 ± 1,3	97,5 ± 3,8
**Ganso**	**MAE**	17,1 ± 2,7 ***	132,2 ± 28,5	45,6 ± 4,9	74,1 ± 1,2 *	283 ± 4,4	41,5 ± 8,6
	**UAE**	69,8 ± 3,9 ns	276,6 ± 8,6	23,1 ± 2,5	82,3 ± 0,7 ns	287,6 ± 2,3	38,5 ± 0,8
**Hallado Alemán**	**MAE**	85,8 ± 0,7 ns	254 ± 17,9	126,3 ± 7,9	81,8 ± 1,2 ns	300 ± 1,8	100,4 ± 9,8
	**UAE**	11 ± 0,9 ***	31,6 ± 0,8	40,1 ± 2,4	73,3 ± 2,9 ns	290,8 ± 2,2	89,8 ± 7,7
**Lunatus**	**MAE**	86,8 ± 0,8 ns	250,6 ± 22,5	78,4 ± 4,1	90,8 ± 0,4 ns	314,4 ± 6,9	62,2 ± 1,1
	**UAE**	7,8 ± 1,4 ***	28,6 ± 1,3	32,5 ± 3,4	72,6 ± 1,6 **	276,6 ± 3,9	103,7 ± 7,6
**Manteca**	**MAE**	55,2 ± 1,3 ***	297,4 ± 0,4	29,3 ± 2,1	79,8 ± 1,4 ns	297,6 ± 0,5	33 ± 1,0
	**UAE**	78,5 ± 2,9 ns	298 ± 0,6	39 ± 1,9	87,9 ± 1,2 ns	295,6 ± 0,6	46,6 ± 1,0
**Mantequilla**	**MAE**	22,3 ± 1,8 ***	34,8 ± 5,9	45,9 ± 5,8	76,7 ± 2,4 ns	327,8 ± 13	77,2 ± 4,9
	**UAE**	56,3 ± 1,3 ***	300 ± 0	26,3 ± 2,0	76,4 ± 1,8 ns	300,8 ± 6,4	57,3 ± 1,8
**Negro**	**MAE**	7,8 ± 1,2 ***	34,8 ± 1,4	35,6 ± 3,2	73,6 ± 2,0 *	284,8 ± 5,1	88,1 ± 6,6
	**UAE**	25,3 ± 1,4 ***	329,4 ± 76,9	70,8 ± 2,6	84 ± 0,9 ns	296,4 ± 0,8	144,5 ± 3,1

**Negro Arauco**	**MAE**	28,5 ± 2,8 ***	83 ± 16,3	63,8 ± 2,9	72,8 ± 2,0 *	297,6 ± 0,8	84,6 ± 8,0
	**UAE**	47 ± 1,7 ***	198,2 ± 0,2	21,9 ± 1,5	78 ± 1,4 ^{ns}	291,8 ± 1,4	47,1 ± 5,6
**Pajarito**	**MAE**	56,7 ± 0,9 ***	298,6 ± 0,3	21,2 ± 1,8	84,1 ± 1,1 ^{ns}	298 ± 0,5	53,2 ± 3,2
	**UAE**	78 ± 2,8 ^{ns}	294 ± 1,9	56,8 ± 4,6	87,7 ± 1,3 ^{ns}	297,6 ± 0,3	60,3 ± 4,8
**Palo**	**MAE**	85,4 ± 0,3 ^{ns}	417 ± 15,5	91,6 ± 4,5	81 ± 3,3 ^{ns}	382,8 ± 19,4	59,2 ± 2,4
	**UAE**	23,5 ± 1,5 ***	249 ± 13	28,2 ± 3,2	71,1 ± 3,4 **	299,2 ± 0,2	60,6 ± 1,6
**Pallar Manchado**	**MAE**	20,9 ± 1,0 ***	29,2 ± 0,6	54,4 ± 5,2	88 ± 1,3 ^{ns}	295,4 ± 0,9	106,6 ± 2,9
	**UAE**	18,7 ± 1,1 ***	298,2 ± 0,4	21 ± 1,3	74 ± 0,6 *	296 ± 1,0	73,6 ± 2,5
**Pallar Morado**	**MAE**	31,9 ± 0,6 ***	70 ± 6,6	66,6 ± 3,4	73,5 ± 2,6*	290,8 ± 0,9	101 ± 2,0
	**UAE**	20,8 ± 1,0 ***	235,4 ± 25,8	30 ± 3,3	68 ± 1,6 ***	296,2 ± 0,8	76,1 ± 1,7
**Peumo**	**MAE**	46,1 ± 6,2 ***	150,4 ± 33,6	70,7 ± 4,0	81,8 ± 1,9 ^{ns}	305,6 ± 4,7	110,6 ± 7,8
	**UAE**	81,7 ± 1,7 ^{ns}	328,4 ± 25,9	45,9 ± 8,3	82,9 ± 0,5 ^{ns}	339 ± 15,9	50,2 ± 2,4
**Rojo**	**MAE**	35,5 ± 2,7 ***	135,6 ± 27,8	70,1 ± 3,5	75 ± 2,0 *	288,2 ± 2,8	110,1 ± 3,5
	**UAE**	57,1 ± 7,5 ***	299,8 ± 12,6	88,8 ± 4,1	69 ± 7,0 ***	287 ± 9,1	106 ± 8,9
**Sapito**	**MAE**	69,6±4,7*	385±19	99±15	69,0±4,8*	308±12	72±11
	**UAE**	79,4 ± 3,0 ^{ns}	297,6 ± 0,7	50,9 ± 5,7	87,8 ± 0,9 ^{ns}	298 ± 0,4	44,3 ± 3,1

**Soja**	**MAE**	22,4 ± 2,8 ***	98,4 ± 20,3	53,7 ± 3,2	75,2 ± 2,2 *	312,6 ± 6,9	93 ± 4,6
	**UAE**	28,1 ± 2,9 ***	28,4 ± 1,1	71 ± 4,7	74 ± 1.9 *	255,2 ± 13,5	62 ± 1,9
**Torcaza**	**MAE**	27,5 ± 2,0 ***	116,2 ± 22,6	54,5 ± 3,0	78,7 ± 3,4 ns	285,2 ± 12,4	50,5 ± 2,5
	**UAE**	48 ± 0,7 ***	253 ± 9,6	44,5 ± 3,1	66,2 ± 3,9 ***	259 ± 11,6	52,7 ± 3,8
**Tórtola**	**MAE**	12 ± 1,3 ***	48,2 ± 7,4	38,1 ± 2,3	78,2 ± 1,7 ns	241,6 ± 23,3	72,1 ± 6,8
	**UAE**	75,6 ± 2,9 ns	298,6 ± 0,5	35,7 ± 3,8	80,8 ± 1,2 ns	294 ± 1,0	40,2 ± 3,1
**Control**		80,5 ± 1,9	301,6 ± 4,5	35,7 ± 3,8	85,1 ± 1,2	299 ± 3,0	49,2 ± 5,1

Data are expressed as mean ± SD, n=5, and analyzed using one-way ANOVA. Post hoc analyzes were performed using the Dunnet test, * $p < 0.05$, ** $p < 0.01$ and *** $p < 0.001$ denotes a statistically significant difference compared to vehicle; ns: non-statistical difference compared to vehicle. AUC: Area under the curve; MAE: Microwave-assisted extraction; PA: Percentage of platelet aggregation; UAE: Ultrasound-assisted extraction.

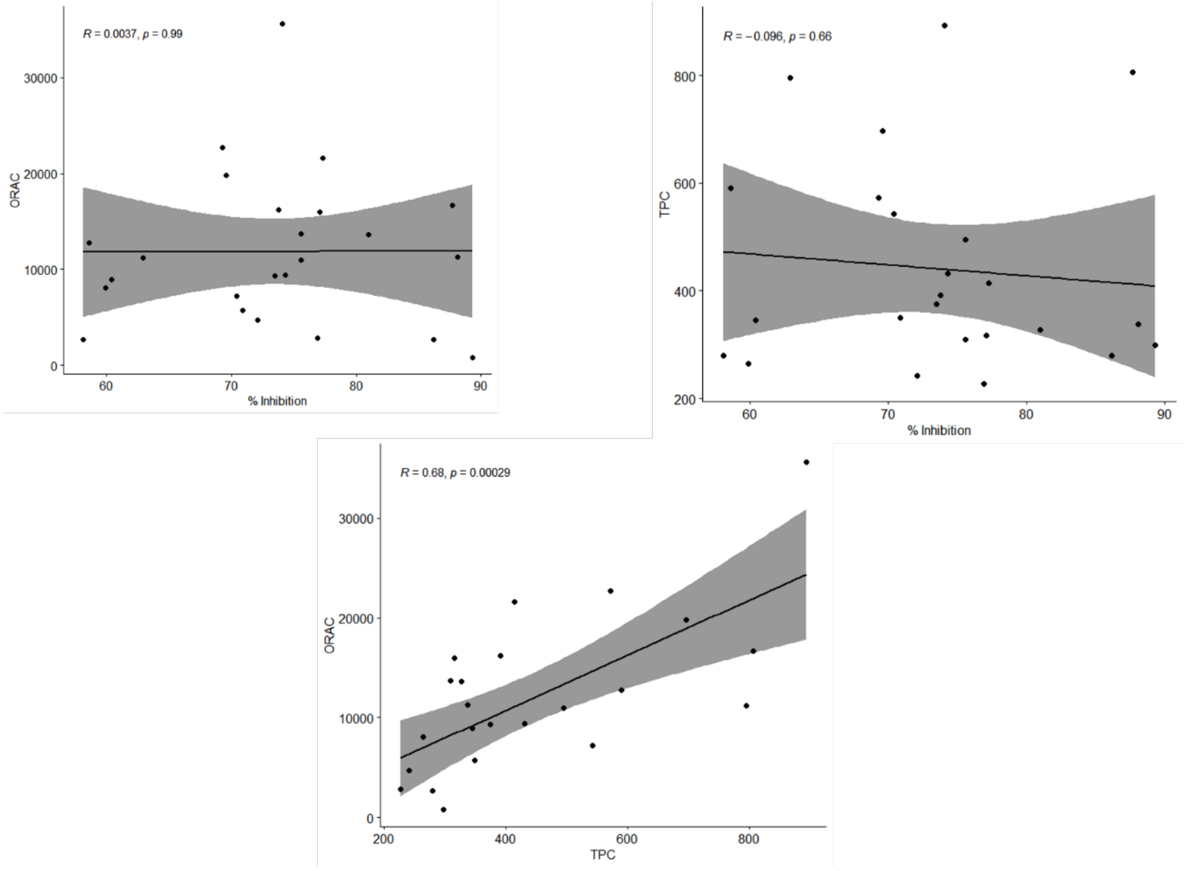

Figure S1. Correlation between phenol content, antioxidant activity, and antiplatelet activity of extracts *P. vulgaris* L.

References

1. Alcázar-Valle, M., et al., *Bioactive compounds, antioxidant activity, and antinutritional content of legumes: a comparison between four phaseolus species.* 2020. **25**(15): p. 3528.
2. Mojica, L., et al., *Bean cultivars (Phaseolus vulgaris L.) have similar high antioxidant capacity, in vitro inhibition of α-amylase and α-glucosidase while diverse phenolic composition and concentration.* 2015. **69**: p. 38-48.
3. Sutivisedsak, N., et al., *Microwave-assisted extraction of phenolics from bean (Phaseolus vulgaris L.).* 2010. **43**(2): p. 516-519.
4. Kumar, M., et al., *Evaluation of enzyme and microwave-assisted conditions on extraction of anthocyanins and total phenolics from black soybean (Glycine max L.) seed coat.* 2019. **135**: p. 1070-1081.
5. Yang, Q.Q., et al., *Polyphenols in common beans (Phaseolus vulgaris L.): Chemistry, analysis, and factors affecting composition.* Comprehensive Reviews in Food Science and Food Safety, 2018. **17**(6): p. 1518-1539.

6. Ombra, M.N., et al., *Phenolic composition and antioxidant and antiproliferative activities of the extracts of twelve common bean (Phaseolus vulgaris L.) endemic ecotypes of Southern Italy before and after cooking.* 2016. **2016**.
7. Singh, B., et al., *Ultrasound assisted extraction of polyphenols and their distribution in whole mung bean, hull and cotyledon.* 2017. **54**(4): p. 921-932.
8. Luo, J., et al., *Phytochemical distribution in hull and cotyledon of adzuki bean (Vigna angularis L.) and mung bean (Vigna radiate L.), and their contribution to antioxidant, anti-inflammatory and anti-diabetic activities.* 2016. **201**: p. 350-360.
